# Polymerase Θ is a key driver of genome evolution and of CRISPR/Cas9-mediated mutagenesis

**DOI:** 10.1038/ncomms8394

**Published:** 2015-06-16

**Authors:** Robin van Schendel, Sophie F. Roerink, Vincent Portegijs, Sander van den Heuvel, Marcel Tijsterman

**Affiliations:** 1 Department of Human Genetics, Leiden University Medical Center, PO Box 9600, 2300 RC, Leiden, The Netherlands; 2 Department of Biology, Division of Developmental Biology, Utrecht University, Padualaan 8, 3584 CH, Utrecht, The Netherlands

## Abstract

Cells are protected from toxic DNA double-stranded breaks (DSBs) by a number of DNA repair mechanisms, including some that are intrinsically error prone, thus resulting in mutations. To what extent these mechanisms contribute to evolutionary diversification remains unknown. Here, we demonstrate that the A-family polymerase theta (POLQ) is a major driver of inheritable genomic alterations in *Caenorhabditis elegans*. Unlike somatic cells, which use non-homologous end joining (NHEJ) to repair DNA transposon-induced DSBs, germ cells use polymerase theta-mediated end joining, a conceptually simple repair mechanism requiring only one nucleotide as a template for repair. Also CRISPR/Cas9-induced genomic changes are exclusively generated through polymerase theta-mediated end joining, refuting a previously assumed requirement for NHEJ in their formation. Finally, through whole-genome sequencing of propagated populations, we show that only POLQ-proficient animals accumulate genomic scars that are abundantly present in genomes of wild *C. elegans*, pointing towards POLQ as a major driver of genome diversification.

## Abstract (web summary)

DNA double-stranded breaks can be repaired through error-prone pathways. Here, van Schendel *et al.* demonstrate that *C. elegans* acquires inheritable mutations through the use of polymerase theta-mediated end joining.

## Graphical abstract

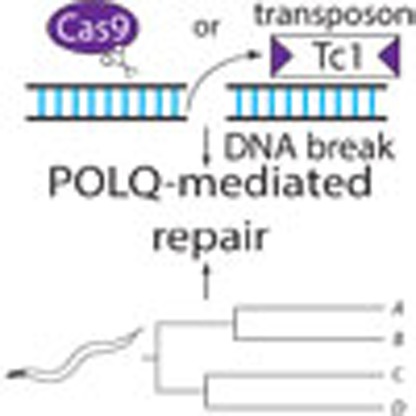

Identifying the mechanisms that drive heritable genome alterations is important for our understanding of carcinogenesis, inborn disease and evolution. Several repair mechanisms exist to avoid the potentially detrimental effects of DNA breaks: homologous recombination (HR) repairs DSBs in an error-free manner, but only when an undamaged template is available; non-homologous end joining (NHEJ) joins the ends of a DNA break without the use of a repair template, frequently resulting in sequence alterations^1^. In addition to these two well-established repair modes, other genetically less-defined mechanisms operate mostly under circumstances that are more rare and incompletely understood. An alternative end-joining (alt-EJ) pathway was described that generally manifests only when NHEJ is compromised^2^,^3^,^4^. The A-family polymerase theta (POLQ) was recently identified to play a major role in alt-EJ of DSBs in *Drosophila*, *Caenorhabditis elegans*, mice and humans^5^,^6^,^7^,^8^,^9^,^10^. Several other functions have been suggested for POLQ, besides operating in alt-EJ, which includes bypassing DNA lesions^11^,^12^,^13^ and influencing the timing of DNA replication origin firing^14^. Mice lacking functional POLQ show a very mild enhanced chromosome instability phenotype, which is exacerbated in combination with a deficiency in ATM, a kinase involved in the repair of DSBs^13^,^15^. The recent discovery that HR-deficient tumours are dependent on repair by POLQ also argues that HR and alt-EJ can act on similar substrates, and importantly identifies POLQ as a druggable candidate target for cancer therapy^5^. The physiologically relevant contexts for when alt-EJ is the repair route of choice are, however, largely unknown. Recent work in *C. elegans* suggested that POLQ is important in repairing replication-associated DSBs in cells that fail to bypass endogenous DNA lesions^9^ or unwind thermodynamically stable DNA structures^6^. Other observations point to the predominance of alt-EJ in germ cells: *de novo* genome deletions and chromotripsis-like chromosome rearrangements underlying congenital disease are frequently characterized by microhomology at their junctions^16^, a feature that has thus far been characteristic for alt-EJ^17^. Such a scenario would also be compatible with the observed lack of expression of key NHEJ proteins during specific (DSB repair-proficient) stages of gametogenesis in vertebrates^18^,^19^. To identify the contribution of DSB repair pathways to inheritable genome change, we studied error-prone repair of DSBs in germ cells of *C. elegans,* and surprisingly found this to be entirely dependent on POLQ-mediated alt-EJ. Moreover, we found POLQ-1 action to be solely responsible for the vast majority of insertion/deletions that occur during natural evolution of *C. elegans*.

## Results

### Transposon breaks are repaired by POLQ-mediated EJ

**Figure 1:**
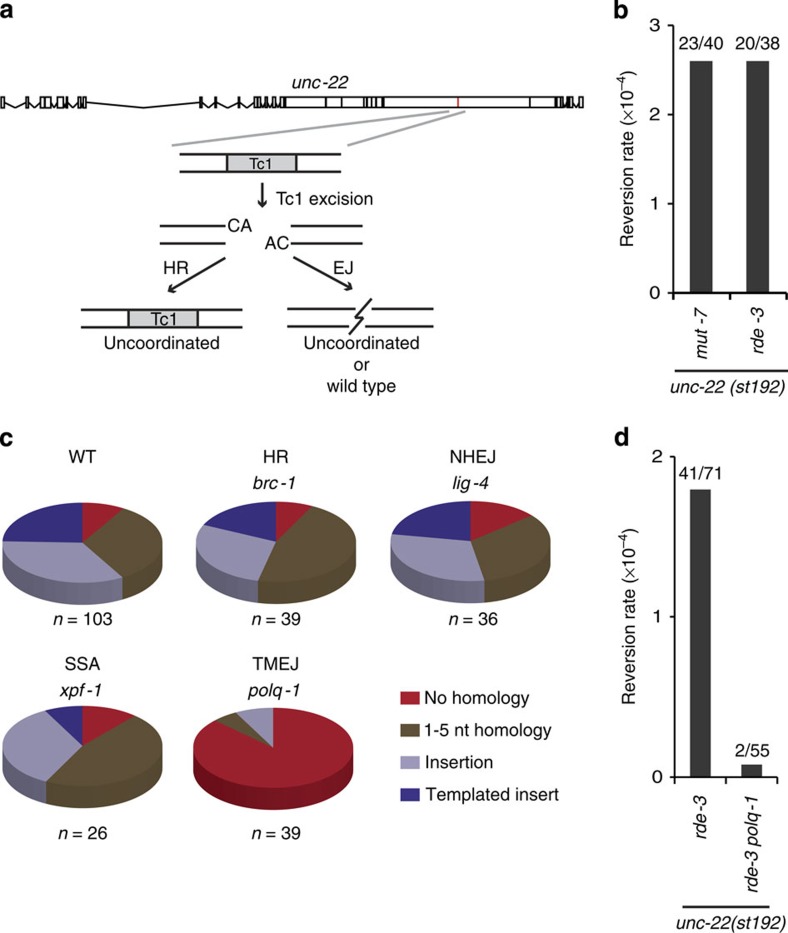
Error-prone repair of transposon-induced DSBs requires POLQ-1. (**a**) Schematic representation of the experimental system to monitor repair of Tc1-induced DSBs. Tc1-encoded transposases can excise a frame-disrupting Tc1 element (*unc-22::st192*) from the endogenous *unc-22* gene, thus resulting in a DSB within the *unc-22* ORF with non-complementary 3′ overhangs of two nucleotides. In case of repair through HR, the original (Tc1-containing) sequence will be restored without affecting the phenotype of progeny cells. Error-prone EJ can lead to *unc-22* ORF correction, which, when occurring in germ cells, will result in wild-type-moving progeny born out of uncoordinatedly moving *unc-22* mutant animals. (**b**) Reversion frequencies of Tc1 for two different genetic backgrounds (*rde-3* and *mut-7*) that de-repress transposon silencing^53^. For each mutant background, ∼20 populations were scored for the presence of revertants and experiments were performed in duplicate. The total number of populations that were assayed and the number of populations that contained at least one revertant animal are indicated. Populations contained, on average, 2,000 animals. (**c**) Distribution of footprints in *unc-22(st192)* for the indicated genomic backgrounds; all strains were also *rde-3* deficient. The number of independently derived reversion alleles is depicted underneath. Distinct footprints (26 in repair-proficient animals) were classified into the following four separate categories: (i) simple deletions without homology at the deletion junction (red), (ii) simple deletions with 1–5 bp of sequence homology at the deletion junction (brown), (iii) deletions that also contained insertions (light blue), and (iv) deletions with associated insertions that were identical to sequences immediate flanking the break (blue). (**d**) Quantification of the *unc-22(st192)* reversion frequency in *rde-3* and *polq-1; rde-3* mutant backgrounds. The number of populations that were assayed and the number of populations that contained at least one revertant animal are indicated. Populations contained, on average, 2,400 animals.

In *C. elegans*, DNA transposons of the Mariner family are a natural source of genome change: upon hopping into a new location, transposons leave behind a DSB that in somatic cells is repaired by NHEJ^20^, but in germ cells it is either repaired error free by HR^21^ or error prone by an EJ mechanism that is currently unknown^20^,^22^. We first inspected the genomes of 45 sequenced natural isolates of *C. elegans*^23^,^24^ for genomic scars associated with DNA transposition. Although we found 93 unique transposon insertions in 23 isolates, too few deletions were identified at known transposon sites (<10) for a systematic analysis of deletion junctions (Supplementary Fig. 1, and Supplementary Data 1 and 2). The high insert versus deletion ratio is in line with previous data arguing that transposon-induced DSBs are predominantly repaired in an error-free manner^21^. To study error-prone repair, we next stimulated DNA transposition under laboratory conditions (by genetically inactivating transposon silencing^25^) and phenotypically monitored DSB repair in germ cells. To this end, animals were used that carry a frame-disrupting Tc1 element in the endogenous *unc-22* gene, which makes them move uncoordinatedly. Tc1 excision followed by imprecise repair of the resulting break can lead to open reading frame (ORF) restoration, and the frequency of wild-type-moving animals in populations of uncoordinated animals thus reflects the frequency of error-prone repair of transposon-induced DSBs in germ cells (Fig. 1a,b). In line with previous findings^22^, we found that NHEJ deficiency did not affect the frequency (2.6E-4 and 2.3E-4, for wild-type and *lig-4* mutant animals, respectively) or pattern of Tc1-induced genomic alterations: in both genetic backgrounds, the spectrum is highly variant, showing 26 distinct deletion products in 103 isolated wild-type animals and 16 distinct footprints in 36 isolated *lig-4* mutant animals (Fig. 1c and Supplementary Data 3). We next found that deficiencies in genes in other DSB repair pathways, that is, HR (*brc-1,* the worm homologue of mammalian breast cancer gene BRCA1) or single-stranded annealing (*xpf-1*/*ercc-1*) also did not affect the mutation spectrum of insertions/deletions (indels) at Tc1-induced breaks (Fig. 1c and Supplementary Fig. 2), nor did defects in mismatch repair or translesion synthesis (Supplementary Fig. 2 and Supplementary Data 4). However, in-depth analysis of >100 deletion footprints derived from wild-type populations provided a strong clue about the identity of the repair process that is responsible for their generation: ∼79% of all deletions that were simple (that lost only the Tc1 element and some flanking nucleotides, *n*=43) displayed single-nucleotide homology, a feature that was recently attributed to the action of an alternative form of end joining (EJ) that critically depends on the A-family polymerase POLQ^6^,^9^. In addition, another described feature of polymerase theta-mediated EJ (TMEJ) stood out in this collection of repair products: 24% of all deletions contained, in addition to the loss of the Tc1 element and a few flanking nucleotides, DNA inserts of which the sequence was identical to sequences in close proximity to the DSB, so-called templated inserts^26^,^27^. Indeed, we found that inactivation of *polq-1*, the gene encoding POLQ, markedly affected the outcome of transposon-induced DSB repair: a profound reduction (>20-fold) in the number of deletion products was observed and also the spectrum of the remaining products greatly changed (Fig. 1c–d). No templated inserts were found, and one class of footprints, which is devoid of single-nucleotide homology and may have been the result of blunt ligation of limitedly processed ends, dominated the spectrum (32 out of 39 repair products). We conclude from these data that TMEJ is responsible for >95% of error-prone repair of transposon-induced breaks in germ cells of *C. elegans*. Reconstructing how individual templated inserts came about (Supplementary Fig. 3) allows us to construct a detailed mechanistic model for TMEJ on DSBs, in which minute base pairing interactions of two 3′ single-strand DNA tails at either side of the break are sufficient to prime DNA synthesis by POLQ-1, leading to a DNA complementarity-driven stabilization of the broken ends.

### POLQ-mediated repair of CRISPR/Cas9-induced breaks

**Figure 2:**
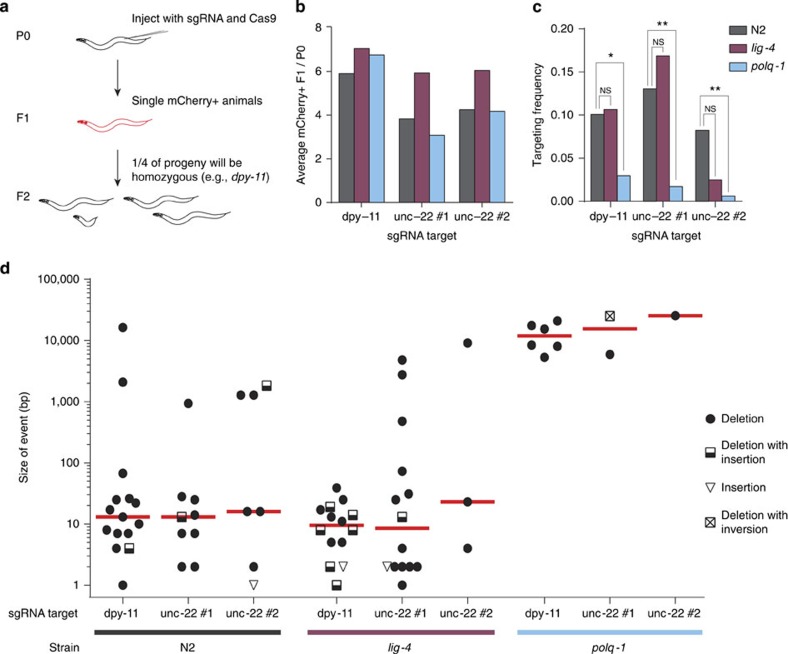
CRISPR/Cas9-induced mutations are generated through TMEJ. (**a**) Schematic illustration of the strategy to generate mutants via CRISPR/Cas9 technology in *C. elegans*. Hermaphroditic animals (P0) are microinjected with plasmids that provide germline expression of Cas9 and of guide RNAs that target genes of interest (*dpy-11* and *unc-22*). A marker plasmid that results in somatic mCherry expression was co-injected. Only mCherry-positive progeny animals (F1) were clonally grown because these have, when compared with non-expressing progeny animals, a higher chance of carrying a (heterozygous) mutation in the targeted gene. Homozygous mutant animals will manifest in a Mendelian manner in the brood (F2) of transformed F1's because of hermaphroditism. (**b**) A quantification of the efficiency of transgenesis in animals of different genotype. The average number of mCherry-expressing animals per injected P0 animal is indicated for each sgRNA target. More than 20 animals were injected per experimental condition. (**c**) A quantification of the efficiency of CRISPR/Cas9-induced gene targeting per sgRNA target in animals of different genotype. The frequency is defined as the number of mutant alleles divided by the number of successfully transformed F1 progeny animals. A Fisher's exact test was used to determine statistical significance. (NS, nonsignificant, *P<0.05, **P<0.01). (**d**) A size representation of CRISPR/Cas9-induced mutants that were obtained in wild-type, *lig-4* and *polq-1* mutant animals. Three different sgRNAs, targeting two genes were used. The median is indicated in red.

To further substantiate this finding and also to look at substrate specificity, we next studied DSBs that were brought about by the clustered, regularly interspersed, short palindromic repeats (CRISPR) RNA-guided Cas9 nuclease^28^. CRISPR/Cas9 technology is used to create mutants in a broad spectrum of biological systems, including worms, flies, fish, plants and mice^29^,^30^,^31^,^32^. The basic principle is to generate a DSB by introducing a guide RNA, which forms a RNA:DNA duplex at a target site, which is then recognized and cut by Cas9. It has been suggested that CRISPR/Cas9-induced breaks are repaired by NHEJ in these systems. However, we here show that CRISPR/Cas9-mediated germline transformation in *C. elegans* is entirely mediated by TMEJ and not by NHEJ. We created mutant animals by microinjecting CRISPR plasmids targeting three sites at two distinct loci into the gonadal syncytium of hermaphroditic *C. elegans (*Fig. 2a). Deletion alleles were generated with ∼10% efficiency per progeny that has been successfully transformed (Fig. 2b,c and Supplementary Table 2). Most of the obtained alleles had a small deletion, with a median size of ∼13 base pairs (bp) for each target (Fig. 2d and Supplementary Data 5). This outcome is in agreement with all currently available worm data on CRISPR alleles, arguing little effect of the target's sequence context or genomic environment on the outcome of repair. We found that inactivation of NHEJ, by disrupting either *lig-4* or *cku-80* (*C. elegans* Ku80) (Fig. 2d and Supplementary Fig. 4), did not change the frequency or the type of genomic alterations, thus ruling out a role for canonical NHEJ in CRISPR/Cas9-mediated germ cell transformation. In contrast, the efficiency of successful CRISPR/Cas9 targeting dropped at least sixfold for all targets in *polq-1*-deficient animals (Fig. 2c). Moreover, the mutants that were obtained in this background had deletions that were ∼1,000-fold larger, ∼10–15 kb on average (Fig. 2d). We thus conclude that TMEJ is responsible for repair of blunt CRISPR/Cas9-induced DSBs in germ cells giving rise to inheritable alleles. Here, as in the processing of transposon-induced breaks, TMEJ action results in a typical signature: 7% of CRISPR/Cas9 breaks are characterized by templated inserts and 80% of simple junctions have single-nucleotide homology (Supplementary Fig. 5). Break ends that are processed by POLQ also appear to be quite stable, as many deletions have their junction exactly at the position where the blunt-end DSB is made and have lost only few base pairs at one of either ends (Supplementary Fig. 4). The demonstration that POLQ acts dominantly in EJ of CRISPR/Cas9-mediated DSBs raises the question whether it also acts to suppress HR-mediated homologous repair of CRISPR/Cas9 breaks. We found, however, with two different target-repair template combinations that homologous targeting is not more efficient in *polq-1* animals (Supplementary Fig. 6).

### POLQ-mediated repair drives genome evolution

**Figure 3:**
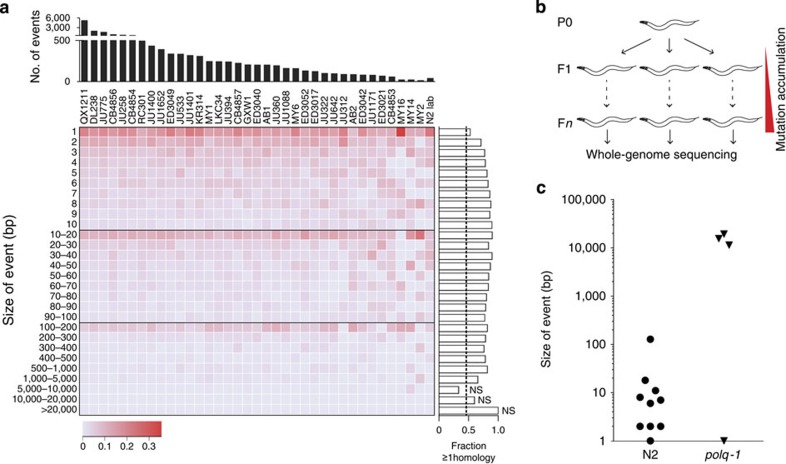
TMEJ is a driver of genomic diversification in C. elegans. (**a**) A heat map representation of all genomic deletions events that were uniquely present in natural isolates of *C. elegans*, in which deletions are binned to size. The intensity of the colour reflects the fraction of deletions in each bin; the number of deletions for each strain is plotted above the heat map. The lane ‘N2 lab' represents deletions that accumulated in the Bristol N2 strains upon culturing in three different laboratories. For each size bin the fraction of microhomology ≥1 is plotted to the right of the heat map. The calculated ratio, as well as an empirically determined ratio, for the presence of microhomology ≥1 is 0.47 for a randomly distributed set of deletions in the *C. elegans* genome^9^, which is represented by a dashed line. All size bins display a statistically elevated level of microhomology (*P*<0.001, binomial test), except for deletions >5,000, which were rare (*n*=19): NS indicates no statistically significant difference to the expected ratio of 0.47. (**b**) Schematic illustration of the experimental setup reflecting small-scale evolution. Progeny animals (F1) from a single hermaphrodite (P0) are picked to separate plates to establish independent populations that were thus isogenic at the start of culturing. To establish bottlenecks and to carefully keep tract of the number of generations (*n*), a small number of progeny animals were transferred to new plates each generation. DNA was isolated from the progeny of a single animal (F*n*) and sequenced by next-generation sequencing technology with a base coverage of ∼30 for each sample. (**c**) A dot plot representing all unique deletion events that were found in the genomes of wild-type (N2) and *polq-1* mutant animals.

Our data reveal a critical role for POLQ in the repair of DSBs in germ cells of *C. elegans*, but does not address the question how relevant TMEJ is for genome change under unperturbed growth. What is the contribution of error-prone DSB repair to genome evolution? We previously found a TMEJ fingerprint in the genomes of *C. elegans* strains that were isolated from different parts of the globe; however, very little could be concluded as to the scale of the involvement, the source of the instability or the possible presence of redundant pathways that may have similar outcomes^9^. Using two complementary approaches, we now provide evidence that TMEJ plays a previously unrecognized major role in genome diversification. First, we sequenced two of the most diverged *C. elegans* strains known, and used these, together with recently sequenced natural isolates of *C. elegans*^23^,^24^, to reconstruct the nature of ∼17,000 unique insertions/deletions (indels). Single-nucleotide variants and indels at microsatellite repeats were excluded from the analysis, as these are likely the product of replication errors and not of error-prone DSB repair. We found the indels in the natural strains to be highly similar to those accumulating in the standard laboratory strain Bristol N2 when grown under laboratory conditions (Fig. 3a). Small deletions (<500 bp), which comprise the vast majority of the indels, had a very similar size distribution in all samples and were characterized by a high degree of single-nucleotide homology at the deletion junctions. Particularly, the latter feature is characteristic for TMEJ of DSBs^6^,^9^. Then, to test whether POLQ is indeed required for the generation of spontaneous indels, we clonally grew wild-type and *polq-1* mutant animals for over 50 generations and then sequenced their genomes (Fig. 3b and Supplementary Table 3). While the induction rate of single-nucleotide variations (SNVs) (0.25 SNVs per generation; Supplementary Fig. 7 and Supplementary Data 6) was identical in wild-type and *polq-1* mutants, the induction rate for deletions was strikingly different: we detected small-sized deletions (median size of 7 bp) only in wild-type animals. This class of mutations was completely absent in the genomes of *polq-1* animals (Fig. 3c, and Supplementary Tables 4 and 5). Instead, extensive deletions (median size of ∼13,500 bp) were found, which *vice versa* were not detected in POLQ-proficient animals, suggesting that in the absence of POLQ the substrates that would induce small deletions are processed differently, thereby leading to massive deletions, which are easily lost from populations because of negative selection. Together, these data argue that the vast majority of indels that are accumulating during nematode evolution is the direct result of POLQ action.

## Discussion

Our data show an unprecedented importance for alt-EJ, which depends on POLQ, in repairing DSBs in the germ cells of *C. elegans*. Previous work has led to the realization that DSBs in *C. elegans* germ cells are either repaired in an error-free manner, through HR, or via an EJ pathway that is different from classical NHEJ^21^,^22^,^33^. We here show that DSBs resulting from transposon mobilization or through the action of the Cas9 endonuclease are repaired via POLQ-mediated EJ, a mechanism that uses single-nucleotide homology and leads to small-sized deletions (of ∼7–13 bp), occasionally accompanied by templated insertions. The reason why NHEJ does not act on these breaks is not known, but it is not because NHEJ is absent from germ cells: we previously demonstrated NHEJ activity on meiotic breaks in animals that were mutated in the worm orthologue of the end-resection factor CtiP^34^. Also, the Fanconi Anaemia pathway has been shown to restrict NHEJ activity in germ cells^35^. An alternative explanation for the inability of NHEJ to process DSBs may be that (restricted) end resection is very efficient in cycling germ cells—early embryonic cell cycles are devoid of recognizable G1 and G2 cell cycle stages—thus leading to 3′ ssDNA overhangs onto which KU70/KU80 complexes do not nucleate a NHEJ reaction. The recent demonstration that POLQ can extend the 3′-hydroxyl end of a 3′-ssDNA tail when minimally paired with another DNA molecule with a 3′-overhang supports the idea that transposon- or Cas9-induced breaks in germ cells are processed to have 3′ overhanging ends^36^. In this scenario, POLQ-mediated EJ repairs DSBs that are processed to feed into HR, but which do not necessarily have an error-free template available, for instance, because the break is introduced before DNA replication, or because both sister chromatids sustain a break. This notion is supported by the recent demonstration that POLQ-mediated repair is very prominent in cases where replication-associated DSBs have unavailable sister chromatids^6^, or in HR-compromised genetic backgrounds^5^,^27^.

We found that POLQ functionality is causally involved in the generation of small indels that are abundantly present in the genomes of wild isolates of *C. elegans*. It argues that physiological DSBs in germ cells are repaired through TMEJ, generating inheritable genome alterations. At present, surprisingly little is known about which mechanisms shape the genome of an animal by generating the mutations onto which natural selection can act. Part of this lack of knowledge is because it is extremely difficult to prove experimentally, even for classes of mutations for which a very likely mechanism has been put forward, such as monotract expansions and contractions through polymerase slippage. Evidence for causality is ideally obtained by witnessing a reduction in mutagenesis upon inactivation of a candidate mechanism. The very low frequency of spontaneous mutagenesis in unperturbed conditions is complicating this issue even further. We mimicked evolution by growing animals for over 50 generation (under laboratory conditions) and then sequenced their entire genome to obtain sufficient data points to address questions concerning spontaneous mutagenesis. We surprisingly found that POLQ is causally involved in the generation of the vast majority of small indels in wild-type animals. This class of indels are also abundantly present in the genomes of wild isolates of *C. elegans*, and our data thus strongly suggest that a mutagenic activity of POLQ is responsible for a major class of genome change during evolution. It is impossible to prove that these indels result from processing of physiological DSBs; however, we consider this very likely because the outcome of POLQ action on programmed DSB is *grosso modo* identical in nature to the indels that accumulate during evolution, with respect to size, use of single-nucleotide homology and the occasional presence of templated inserts. In the absence of POLQ, the mutagenic outcomes are far worse, that is, deletions are ∼1,000-fold larger in size. POLQ thus acts to protect cells but with a small price that manifest as small-sized genomic scars. Which DNA repair pathway is responsible for generating the sizable deletions manifesting in POLQ deficient genetic backgrounds will be the subject of further investigation—the deletion junctions are not characterized by extensive use of homology, which disfavours single-stranded annealing acting as a redundant and mutagenic mechanism to process DSBs.

Surprisingly, on an organismal level, only mild phenotypes result from the absence of POLQ: mice develop normally and are fertile, with a slightly elevated level of genome instability and a subtle, but distinct, reduction in antibody diversification^10^,^15^. Whether POLQ is also a natural driver of genome variation in human germ cells or (cancerous) somatic cells sustaining cell viability at the expense of mutation induction is yet unknown, but the presence of microhomology and the occasional presence of template inserts at junctions of copy number variations, deletions and translocations, as well as in junctions observed in chromotripsis^16^,^37^,^38^ supports such a scenario. Therefore, inhibiting POLQ may, apart from sensitizing cells towards replication stress^9^, restrict the adaptive response of oncogenically transformed cells and thus impair cancer maturation^5^,^39^.

## Methods

### *C. elegans* genetics

Nematodes were cultured on standard NGM plates at 20 degrees^40^. The following alleles were used in this study: *rde-3* (ne298), *mut-7* (pk204), *unc-22* (st192::Tc1), *lig-4* (ok716), *xpf-1* (e1487), *ercc-1* (tm2073), *brc-1* (tm1145), *exo-1*(tm1842), *mlh-1*(gl516), *polh-1*(lf31), *polq-1* (tm2026) and *cku-80* (rb964).

### Reversion assay to identify mutations by Tc1 transposition

Animals carrying *unc-22* (st192::Tc1), *rde-3*(ne298) or *mut-7*(pk204), and wild-type or mutant alleles of DNA repair genes were cultured, keeping track of the presence of the transposon in *unc-22* by selecting for worms that are Unc and by PCR analysis diagnostic for *unc-22*::Tc1. To assay error-prone repair of a DSB at the endogenous *unc-22* locus, single animals were transferred to 6 cm agar plates seeded with OP50 and propagated until starvation. Each experiment typically contained 30–50 plates per genotype. Plates were inspected for the presence or absence of non-Unc wild-type-moving revertants. The reversion frequency is calculated by assuming a Poisson distribution for reversion^41^: Reversion frequency=-ln(*P*_0_)/2*n*, where *P*_0_ is the fraction of plates that did not yield revertants, and *n* is the number of animals that were screened per plate. From plates containing revertant animals, one non-Unc animal was transferred to a new plate and the molecular nature of the events that restored UNC-22 function were determined by PCR analysis and Sanger sequencing on DNA isolated from their brood.

### CRISPR/Cas9-induced mutations and HR

Plasmids were injected using standard *C. elegans* microinjection procedures. Briefly, 1 day before injection, L4 animals were transferred to new plates and cultured at 15 degrees. Gonads of young adults were injected with a solution containing 20 ng μl^−1^ pDD162 (Peft-3::Cas9, Addgene 47549; ref. 42), 20 ng μl^−1^ pMB70 (u6::sgRNA with appropriate target (Supplementary Table 1)), 60 ng μl^−1^ pBluescript, 10 ng μl^−1^ pGH8, 2.5 ng μl^−1^ pCFJ90 and 5 ng μl^−1^ pCFJ104. Progeny animals that express mCherry were picked to new plates 3–4 days post injection. The progeny of these animals was inspected for Mendelian segregation of the corresponding phenotype. For gene targeting through HR, the following injection mix was used: 30 ng μl^−1^ Peft-3::Cas9 (Addgene 46168; ref. 43), 100 ng μl^−1^ pMB70 (u6::sgRNA with appropriate target for *gpr-1* and *lin-5*), 30 ng μl^−1^ HDR template (pVP042 or pVP048), 10 ng μl^−1^ pGH8, 2.5 ng μl^−1^ pCFJ90, 5 ng μl^−1^ pCFJ104. PCRs with primers diagnostic for HR products at the endogenous locus were performed on F2 populations, where one primer resided in the repair template and the other just outside the homology arm (pVP042 GFP Fw: 5′-GAGAGAGGCGTGAAACACAAAG-3′, Rv: 5′-TTTGGGAAGGTACGTCCGTC-3′ 1,796 bp product or pVP048 Fw: 5′-GGCGCATGCACATAATCTTTCA-3′, Rv: 5′-CCAGTGAGCTGCTCTTGAAGA-3′ 1,610 bp product). See Supplementary Data 5 and Supplementary Tables 1 and 2 for more details.

### Plasmid construction

pVP042 was generated to insert sequences encoding an N-terminal protein tag (FKBP-eGFP) into the endogenous *gpr-1* locus. DNA fragments were inserted into the pBSK vector using Gibson Assembly (New England Biolabs). Homologous arms of 1,650 bp upstream and 1,573 bp downstream of the *gpr-1* cleavage site were amplified from genomic DNA using KOD polymerase (Novagen). Codon-optimized FKBP was synthesized (Integrated DNA technologies) and codon-optimized enhanced green fluorescent protein (eGFP) was amplified from pMA-eGFP (a kind gift of Anthony Hyman), and inserted directly 5′ of the ATG of *gpr-1*. Five mismatches were introduced in the sgRNA target site to prevent cleavage of knock-in alleles. pVP048 was generated to alter a single codon in the endogenous *lin-5* coding sequences. DNA fragments were inserted into the pBSK vector using Gibson Assembly (New England Biolabs). Homologous arms of 1,568 bp upstream and 1,557 bp downstream of the *lin-5* cleavage site were amplified from cosmid C03G3 using KOD polymerase (Novagen), a linker containing the altered cleavage site was synthesized (Integrated DNA Technologies). Seven mismatches were introduced in the sgRNA target site to prevent cleavage of knock-in alleles.

### 4,6-diamidino-2-phenylindole staining

L4 worms were picked and allowed to age for 20–24 h. Gonad dissection was carried out in 1 × egg buffer (25 mM HEPES-Cl (pH 7.4), 118 mM NaCl, 48 mM KCl, 2 mM CaCl_2_, 2 mM MgCl_2_, 0.1% Tween-20 and 20 mM sodium azide). An equal volume of 4% formaldehyde in egg buffer was added (final concentration is 2% formaldehyde) and allowed to incubate for 5 min. The dissected worms were freeze-cracked in liquid nitrogen for 10 min, incubated in methanol at −20 °C for 10 min, transferred to PBS/0.1% Tween (PBST), washed 3 × 10 min in PBS/1% Triton X-100 and stained 10 min in 0.5 μg ml^−1^ 4,6-diamidino-2-phenylindole/PBST. Finally samples were de-stained in PBST for 1 h and mounted with Vectashield. Gonads were analysed using Leica DM6000 microscope.

### Small-scale evolution and bioinformatic analysis

Mutation accumulation lines were generated by cloning out F1 animals from one hermaphrodite. Each generation, about three worms, were transferred to new plates. MA lines were maintained for 50–60 generations. Single animals were then cloned out and propagated to obtain full plates for DNA isolation. Worms were washed off with M9 and incubated for 2 h while shaking to remove bacteria from the intestines. Genomic DNA was isolated using a Blood and Tissue Culture Kit (Qiagen). DNA was sequenced on a Illumina HiSeq2000 machine according to manufacturer's protocol. Image analysis, base calling and error calibration were performed using standard Illumina software. Raw reads were mapped to the *C. elegans* reference genome (Wormbase release 235) by BWA^44^. SAMtools^45^ was used for SNV and small indel calling, with BAQ calculation turned off. To identify larger indels and microsatellites, GATK^46^ and Pindel^47^ were used. In cases that only one of the software identified the structural variation, visual inspection was carried out using IGV^48^. Variations were marked as true if covered by both forward and reverse reads, and at least five times covered, while no reads were found that supported the reference genome while all other samples of the identical genotype supported the reference genome. For the analysis of natural isolates, the same criteria were used, but the output was restricted to Pindel and only unique calls were included. In addition, deletions were only included when showing a >3-fold coverage drop of the deleted sequence, but normal coverage in at least five other natural isolates. All sequencing data, including the natural isolates DL238 and QX1211, have been submitted to the NCBI Sequence Read Archive (SRA) with accession ID (SRP046600). Two sequenced N2 strains can be found at accession ID (SRP020555). Genome sequences of other *C. elegans* natural isolates were obtained from refs 23, 24; the genome sequence of PX174 is identical to RC301 (ref. 49) and was excluded from the analysis. The genome of different cultures of N2 were derived from the National Institute of Genetics Japan (NCBI SRA: DRP001005) from the 50 Helminth Genome Initiative (submitted by the Sanger Center, NCBI SRA: ERX278110) and our own data (SRP020555 and SRP046600).

### Transposon evolution

RetroSeq^50^ was used to find genomic positions of transposons that are not present in the *C. elegans* reference genome (WB235). RetroSeq discovery was run in align mode, using a transposon reference file containing all known Tc/mariner-like transposons. A custom script was written to identify those locations that showed hallmarks of a transposon insertion, which is duplication of a flanking TA or TCA sequence, interrupted by a novel DNA sequence (indicative of an insertion). Once a position was identified in one natural isolate, all other natural isolates were analysed. Occasionally, RetroSeq was unable to identify the specific type of transposon. In those cases, >1 possible transposon was assigned to that location. To identify potential transposon deletions, Pindel was used in which ≥8 supporting reads were set as a threshold and 0 reads should support the reference genome. The majority of the deletions were present in multiple natural isolates and were excluded from the analysis, as these likely represent transposon insertions in the lineage that include the reference genome.

### Phylogenetic tree

The phylogenetic tree was created using high-quality SNV calls (SNV quality score ≥100) throughout all natural isolates with ≥5 reads (and >80% of the reads supporting the SNV), and supported by both forward and reverse reads. These criteria applied to the genomes of 44 natural isolates and N2, and resulted in 565,662 SNVs. PLINK^51^ was used for pruning pairs with *r*^2^>0.3 in a sliding 50-marker window at 5-marker steps and minor allele frequency SNPs were filtered out (<0.05), leaving 22,487 informative SNPs. SNPhylo^52^ was subsequently used to create the phylogenetic tree. Bootstrap analysis was performed 1,000 times to determine the reliability of each branch in the tree.

## Additional information

**Accession codes:** Raw sequences have been made publicly available at NCBI SRA (Accession code SRP046600).

**How to cite this article:** van Schendel, R. *et al.* Polymerase Θ is a key driver of genome evolution and of CRISPR/Cas9-mediated mutagenesis. *Nat. Commun.* 6:7394 doi: 10.1038/ncomms8394 (2015).

## Supplementary Material

Supplementary Figures and Tables Supplementary Figures 1-7 and Supplementary Tables 1-5

Supplementary Data 1 Transposon Insertions in C. elegans natural isolates

Supplementary Data 2 Transposon Deletions in C. elegans natural isolates

Supplementary Data 3 Transposon unc-22(st192) reversion events

Supplementary Data 4 Transposon unc-22(st192) reversion events, remaining genotypes

Supplementary Data 5 CRISPR induced mutations

Supplementary Data 6 Whole Genome Sequencing SNVs
