# Supplementary material for: Polymerase Θ is a key driver of genome evolution and of CRISPR/Cas9-mediated mutagenesis
Source: Nat Commun. 2015 Jun 16;6:7394. doi: 10.1038/ncomms8394 (PMC4490562; doi:10.1038/ncomms8394)
Supplement: Supplementary Figures and Tables — Supplementary Figures 1-7 and Supplementary Tables 1-5 [file ncomms8394-s1.pdf]

Supplementary Figure 1

a

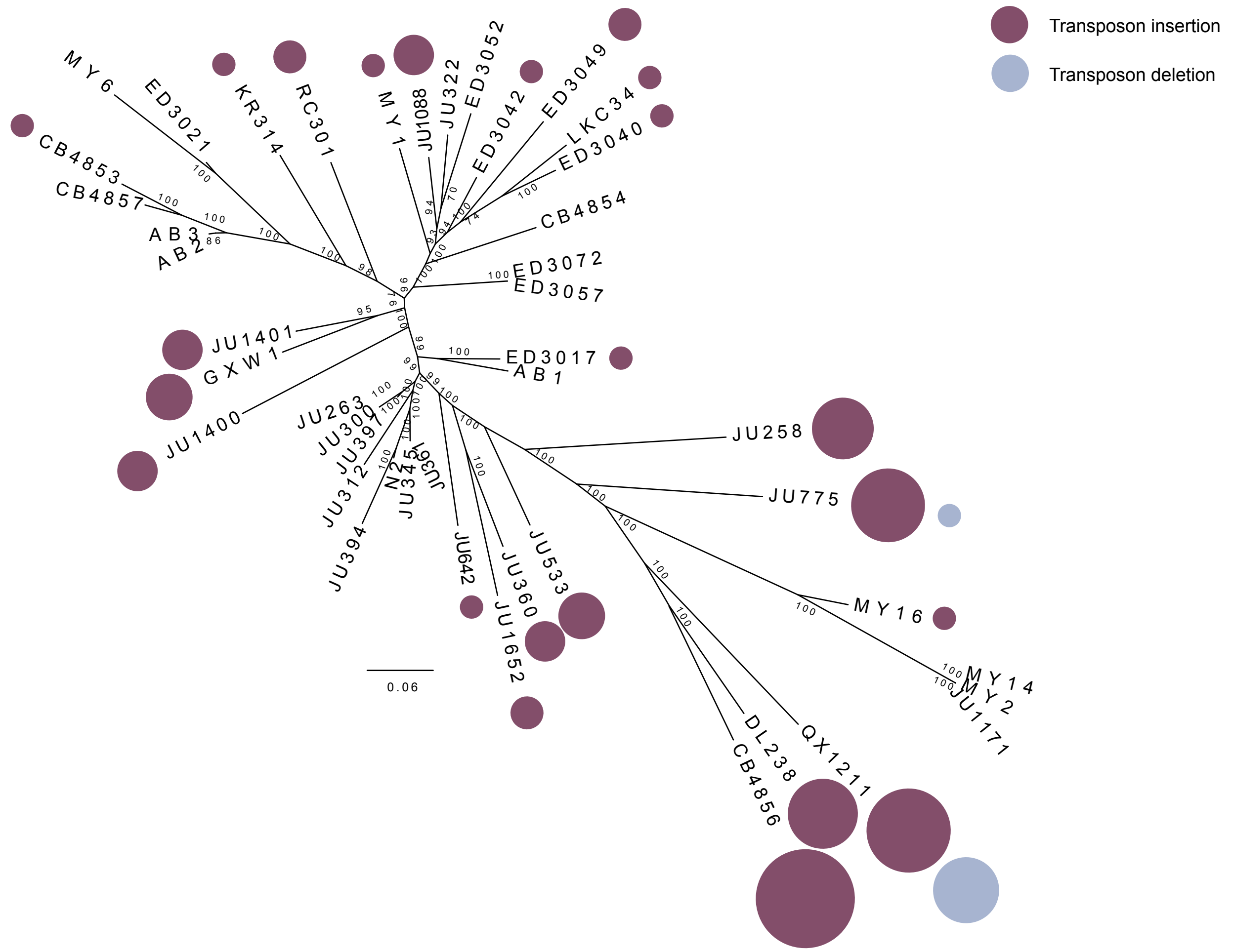

b

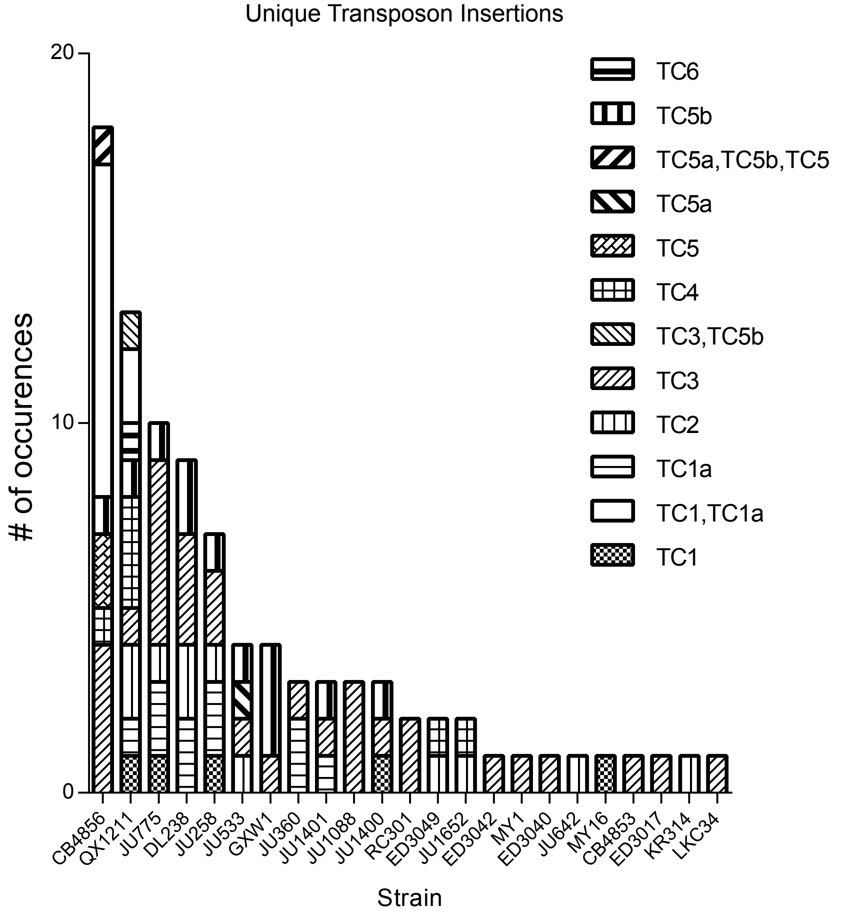

c

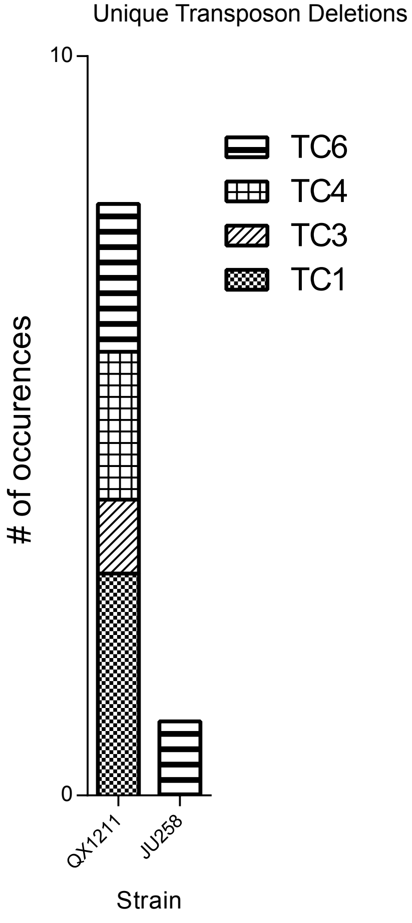

Supplementary Figure 1

DNA transposition in natural isolates of *C. elegans*. A. A phylogenetic tree was constructed from ~22,000 informative SNPs (See Material & Method section for details) present in 45 natural isolates. The outcomes of standard bootstrap analysis (1000 times) are plotted for each branch point. Transposon insertions were identified by RetroSeq, which was specifically designed to find such events in paired-end sequence data. The surface area of the plotted circle reflects the number of insertions (purple) and potential deletions (blue) that are unique to each strain: because the tree is unrooted, it cannot be concluded from this analysis whether the events that are marked as deletions (blue) in QX1211, one of the most diverged strains, are not in fact de novo insertions in a parent-of-origin that spawned all isolates after a split with QX1211. B. The number of unique insertions per type of transposon in each strain is plotted. C. The number of copies per type of transposon that was uniquely absent in each strain is plotted.

Supplementary Figure 2

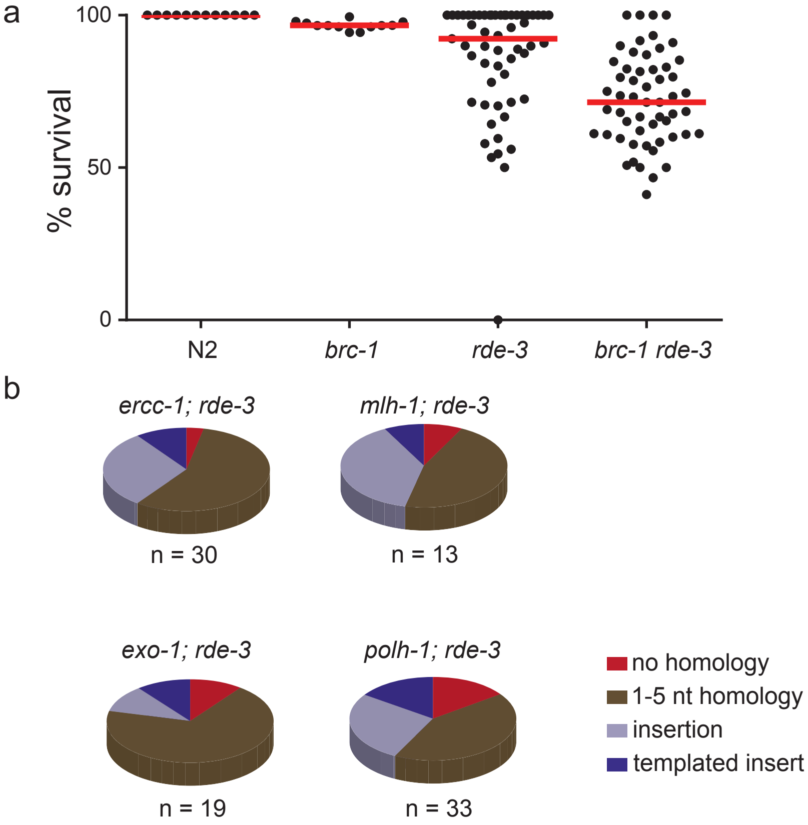

Supplementary Figure 2

Genetic analysis of error-prone repair of transposon-induced breaks in *C. elegans* germ cells. **A.** Transposon breaks-induced embryonic death. Bee swarm plot in which embryo survival is plotted for strains in which transposition is silenced (N2) or de-repressed in germ cells (*rde-3*) and are either proficient or deficient for the homologous recombination gene *brc-1*. Each dot represents the offspring of one animal; the percentage is calculated as the number of hatched larvae divided by the number of total eggs laid. For both N2 and *brc-1*-deficient animals the survival of at least 10 P0 animals was scored, while for the *rde-3*-deficient strains at least 50 P0 animals was scored. The red line represents the median survival for each strain. **B.** Distribution of footprints in *unc-22(st192)* for the indicated genomic backgrounds. The number of independently derived reversion alleles is depicted underneath. Distinct footprints were classified into 4 separate categories: i) simple deletions without homology at the deletion junction (red), ii) simple deletions with 1-5 bp of sequence homology at the deletion junction (brown), iii) deletions that also contained insertions (light blue), and iv) deletions with associated insertions that were identical to sequences immediate flanking the break (blue).

Supplemental Figure 3

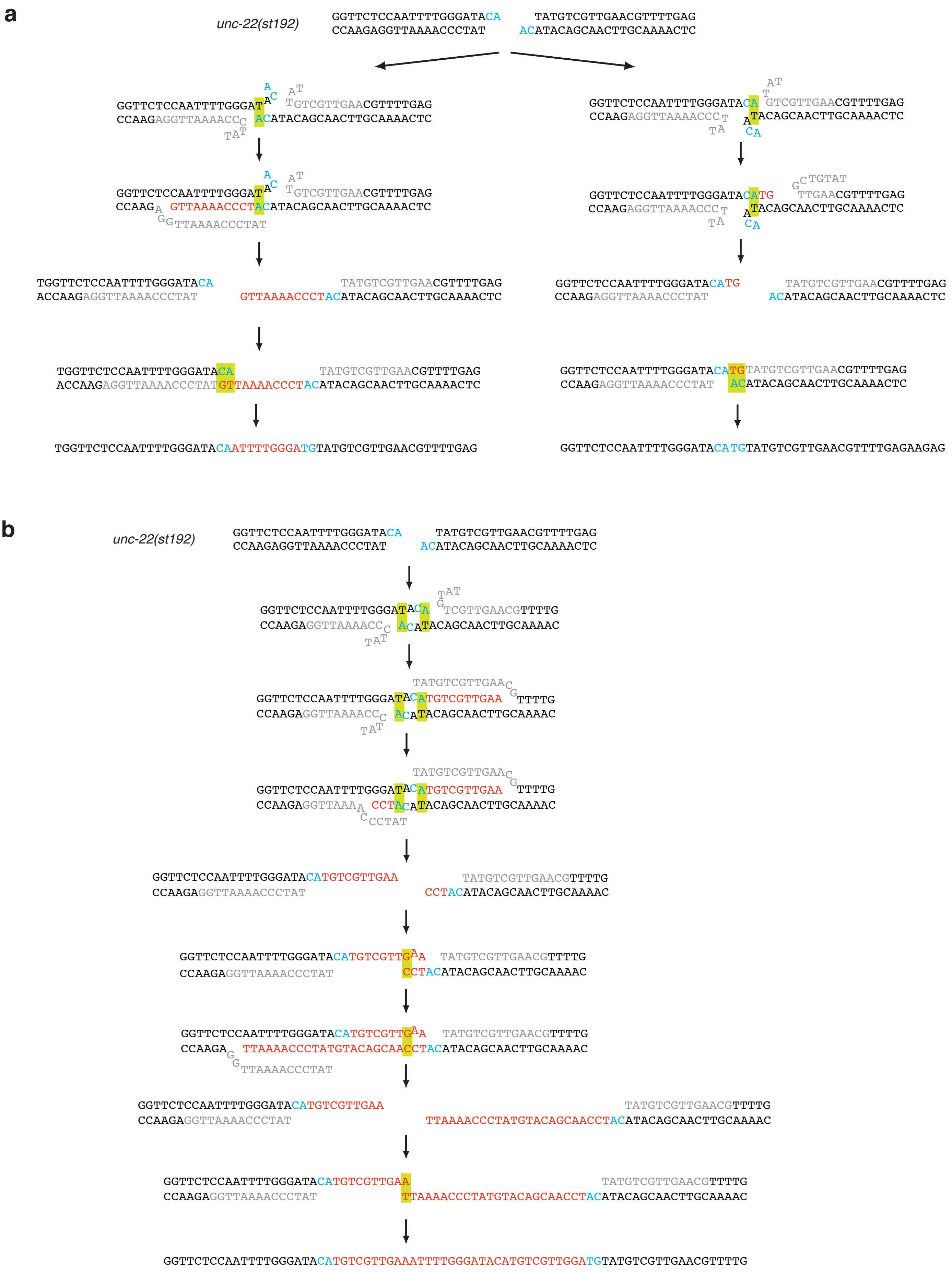

Supplementary Figure 3

Molecular model for TMEJ-generated templated inserts. **A.** Schematic illustration of the consecutive steps of TMEJ of a Tc1-induced DSB leading to the most commonly found templated inserts (12/103 for the outcome displayed on the left; 24/103 for the outcome displayed on the right). The sequence context of *unc-22(st192)* upon excision of Tc1 is displayed, with the 3'CA overhangs in blue. In the first round of the cycle the outermost 3' base (A) has served as a primer for POLQ action by base-pairing (boxed in yellow) to the first available T of the opposite flank; the left flank in the left panel and the right flank in the right panel. Newly synthesized DNA, through the action of POLQ, is displayed in red. Nucleotides that are either displaced by POLQ action or absent because of DSB processing prior to POLQ action are depicted in grey. The formation of the resulting intermediate, that is presumably energetically more stable because of the extended base-pairing of the newly synthesized DNA to its template, is apparently not always driving the process into the generation of simple deletions (without insertions, but with single nucleotide homology). Instead, for thus far unknown reasons, further extension is abrogated, and subsequently the outmost 3' base will search for a new match to re-anneal and again serve as a primer in a second attempt to join both ends. It is noteworthy that the most prominent templated inserts (left and right panel) are conceptually identical: in the first cycle DNA synthesis is continued up to the point where the two outermost 3' nucleotide of newly synthesized DNA can base-pair with the outermost 2 nucleotides of the template strand. **B.** Re-iteration of the steps displayed in A can explain even the most complex inserts. In the illustrated case, both flanks served as template for DNA synthesis; the left flank 3 times and the right flank 2 times, and all DNA synthesis events were primed with 1 nt base pairing.

Supplementary Figure 4

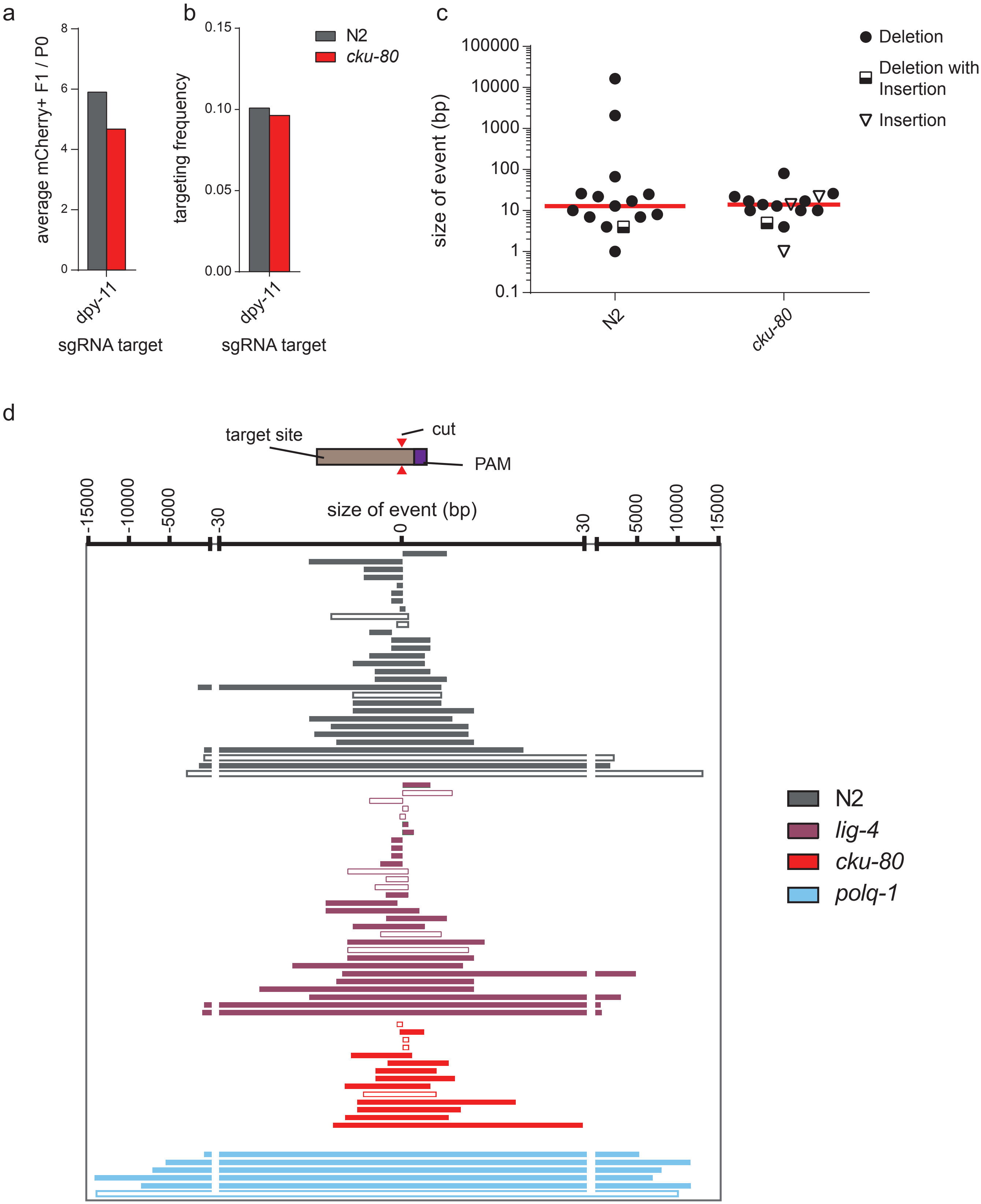

Supplementary Figure 4

Genetic and molecular analsyis of CRISPR/Cas9-induced genome rearrangements. **A-C.** Error-prone repair of CRISPR/Cas9-induced DSBs is independent of NHEJ protein CKU-80. **A.** A quantification of the efficiency of transgenesis in wild type (N2) and *cku-80*-deficient animals. The average number of mCherry-expressing animals per injected P0 animal is indicated. At least 20 animals were injected per strain. **B.** A quantification of the efficiency of CRISPR/Cas9-induced gene targeting of the *dpy-11* locus in wild type (N2) animals and *cku-80* deficient animals. The frequency is the number of mutant alleles divided by the number of successfully transformed F1 progeny animals. **C.** A size representation of CRISPR/Cas9-induced *dpy-11* mutants that were obtained in wild type and in *cku-80* mutant animals. The median is indicated in red. **D**. A visual representation of the CRISPR/Cas9-induced *dpy-11*, *unc-22* (target 1) and *unc-22* (target 2) alleles that were obtained in the strains of indicated genotype. 0 (bp) defines the cut-site of the sgRNA/Cas9 complex, and the orientation of the target and PAM site relative to 0 is depicted. Bars represent the DNA sequence that is lost in each allele. Closed bars represent simple deletions; open bars represent insertions, deletion with insertions and deletions with inversions.

Supplementary Figure 5

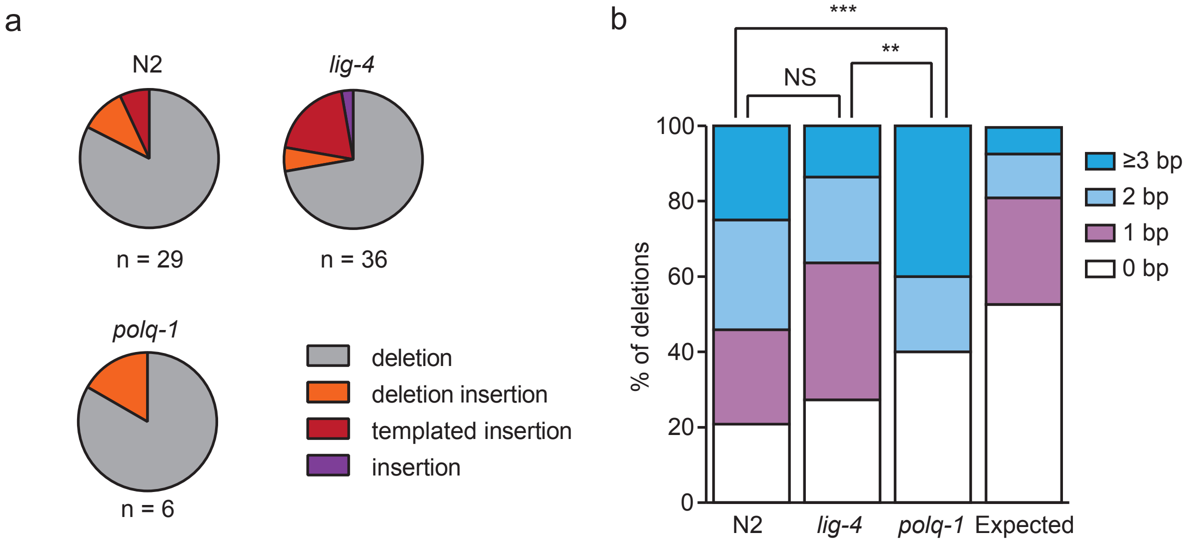

Supplementary Figure 5

Types and homology distribution of CRISPR/Cas9 induced mutations. **A.** The distribution of mutational classes in strains of indicated genotype. **B.** Quantification of the extent of microhomology for the simple deletions obtained in strains of indicated genotype. The distribution that is expected if deletions were randomly distributed is also indicated. The distribution in wild type (N2) and *lig-4* mutant animals is statistically not significantly different (NS), however, both are different from *polq-1*. (** $p < 0.01$, *** $p < 0.001$, T-test)

Supplementary Figure 6

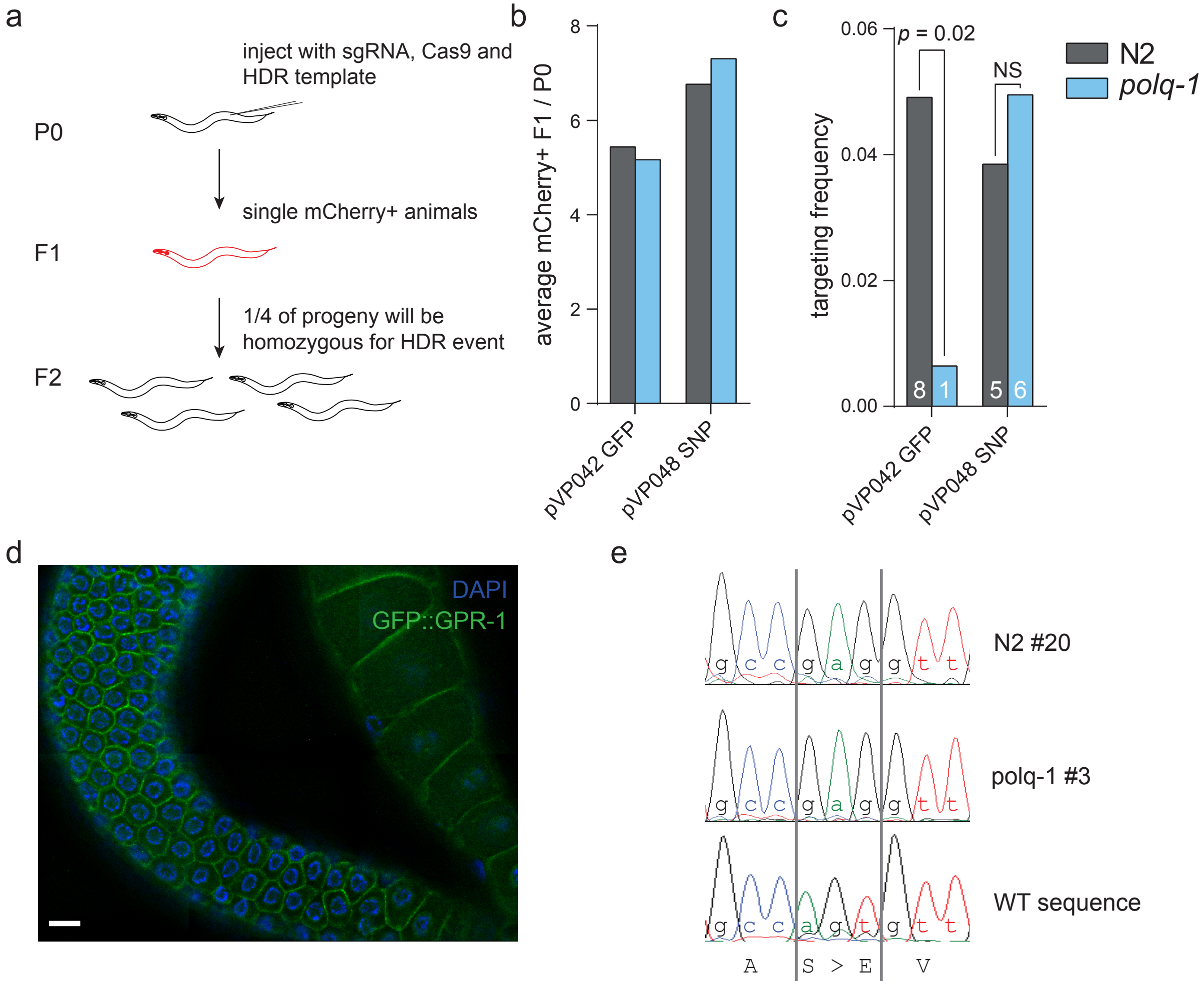

Supplementary Figure 6

No increase in Homology Directed Repair (HDR) in animals defective for POLQ-1 **A.** A schematic illustration of the strategy to generate CRISPR/Cas9-induced alleles via HDR in *C. elegans*. Hermaphroditic animals (P0) are microinjected with a plasmid that provides germline expression of Cas9, a guide RNA that targets a gene of interest, and a plasmid that has a template for HDR. A marker plasmid that results in somatic mCherry expression is also co-injected. Only mCherry-positive progeny animals (F1) were clonally grown because these have, when compared to non-expressing progeny animals, a higher chance of carrying a (heterozygous) mutation in the targeted gene. Homozygous mutant animals will manifest in a Mendelian manner in the brood (F2) of transformed F1's because of hermaphroditism. **B.** A quantification of the efficiency of transgenesis in animals of different genotype. The average number of mCherry-expressing animals per injected P0 animal is indicated for each target locus. At least 20 animals were injected per target per strain. **C.** A quantification of the efficiency of CRISPR/Cas9-induced gene targeting in wild type and *polq-1* mutant animals for the indicated locus. The frequency is the number of mutant alleles divided by the number of successfully transformed F1 progeny animals. A Fisher's exact test was used to determine statistical significance**. D.** Representative image of successful HDR-mediated targeting of GFP to the endogenous *gpr-1* locus using CRISPR/Cas9. GPR-1::GFP (green) expression is visible in the cortex of germ cells in the distal (left) and proximal (right) area of the gonadal syncytium. DAPI staining in blue marks nuclei. Scale bar = 5 µm. **E.** Sanger sequences of two SNP alleles that were generated via CRISPR/Cas9-induced HDR. The wild type and mutant DNA as well as the amino acid sequence is indicated.

Supplementary Figure 7

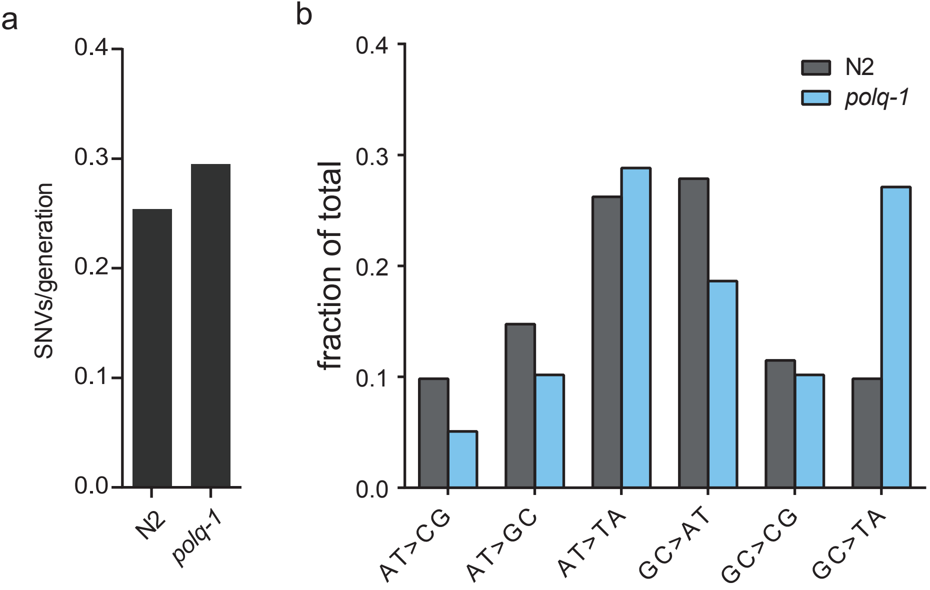

Supplementary Figure 7

Comparable SNV induction rates and distributions in wild type (N2) and *polq-1* mutant animals. **A.** Quantification of the SNV induction rate (SNVs per generation) for the indicated genetic background. The data were obtained from sequencing 4 times 60 generations of wild type growth and 4 times 50 generations of polq-1 growth. **B.** The base composition of the SNVs that accumulated in wild type and polq-1 mutant animals. Both distributions are comparable, apart from an elevated rate of GC>TA mutations in POLQ-1 deficient animals.

Supplementary Table 1. CRISPR targets

Target	Sequence	Chromosome	Start	End
dpy-11	GCAAGGATCTTCAAAAAGCATGG	CHROMOSOME_V	6512819	6512842
unc-22 #1	GACTGCTTGCGGAGAGAGCAAGG	CHROMOSOME_IV	11985320	11985343
unc-22 #2	GAAAAGCAAGATGCTGCCACTGG	CHROMOSOME_IV	11985349	11985372

Supplementary Table 2. CRISPR injections

Strain	Allele	Target	Injected P0s	mCherry+F1	Targeting succes
N2		dpy-11	20	118	12
RB873	lig-4 (ok716)	dpy-11	20	141	16
XF152	polq-1 (tm2026)	dpy-11	37	250	8
N2		unc-22 #1	18	69	9
RB873	lig-4 (ok716)	unc-22 #1	14	83	14
XF152	polq-1 (tm2026)	unc-22 #1	38	117	2
N2		unc-22 #2	20	85	7
RB873	lig-4 (ok716)	unc-22 #2	20	121	3
XF152	polq-1 (tm2026)	unc-22 #2	40	167	1
N2		HDR #1 GFP	30	163	8
XF152	polq-1 (tm2026)	HDR #1 GFP	30	155	1
N2		HDR #2 SNP	23	168	6
XF152	polq-1 (tm2026)	HDR #2 SNP	17	115	5
RB964	cku-80 (ok861)	dpy-11	40	187	18

Supplementary Table 3. Whole genome sequence information

Sample_ID	Allele	#Generations	Average Coverage
N2_2	N2	60	28x
N2_3	N2	60	63x
N2_4	N2	60	16x
N2_50	N2	60	27x
XF151_E50	polq-1 (tm2026)	50	31x
XF151_G50	polq-1 (tm2026)	50	18x
XF151_H50	polq-1 (tm2026)	50	38x
XF151_I50	polq-1 (tm2026)	50	44x

Supplementary Table 4. CNVs

SampleGroup	Sample	Chr	Size	Start	End
N2	N2_3	CHROMOSOME_III	128	1407845	1407973
N2	N-4	CHROMOSOME_I	18	4336437	4336455
N2	N-2	CHROMOSOME_X	11	16256644	16256655
N2	N-4	CHROMOSOME_I	8	7667695	7667695
N2	N-2	CHROMOSOME_II	7	10938339	10938346
N2	N-2	CHROMOSOME_V	6	18324033	18324039
N2	N2_50	CHROMOSOME_V	2	12400541	12400543
N2	N2_3	CHROMOSOME_II	2	13447058	13447060
N2	N-4	CHROMOSOME_II	2	11519211	11519213
N2	N-2	CHROMOSOME_I	1	4524154	4524155
polq-1	XF152_I50	CHROMOSOME_IV	19397	8038373	8057770
polq-1	XF152_E50	CHROMOSOME_V	15588	15466490	15482078
polq-1	XF152_E50	CHROMOSOME_V	11424	3111176	3122600
polq-1	XF152_E50	CHROMOSOME_II	1	1517738	1517738

Supplementary Table 5. Microsatellite changes

SampleGroup	Sample	Chr	Size	Start	End	Change	TractType	TractString
N2	N2_3	CHROMOSOME_IV	3	1377786	1377789	DEL	MONO	gggGGGGGGGGGG
N2	N2_3	CHROMOSOME_X	3	2042489	2042492	DEL	MONO	gggGGGGGGGGGGGGGG
N2	N2_3	CHROMOSOME_X	2	3470709	3470711	DEL	MONO	ggGGGGGGGGGGGG
N2	N2_50	CHROMOSOME_II	1	240972	240972	SINS	MONO	aAAAAAAAAA
N2	N2_3	CHROMOSOME_II	1	3409898	3409899	DEL	MONO	tTTTTTTTT
N2	N2_50	CHROMOSOME_IV	1	3679599	3679600	DEL	MONO	gGGGGGGGGGG
N2	N2_3	CHROMOSOME_III	1	4430962	4430963	DEL	MONO	aAAAAAA
N2	N2_3	CHROMOSOME_I	1	6149746	6149747	DEL	MONO	tTTTT
N2	N2_3	CHROMOSOME_X	1	8972343	8972343	SINS	MONO	tTTTTTTTTTT
N2	N2_50	CHROMOSOME_II	1	10591087	10591088	DEL	MONO	tTTTTTTTTTT
N2	N2_3	CHROMOSOME_III	1	11300284	11300285	DEL	MONO	tTTTTTTTT
N2	N2_50	CHROMOSOME_I	1	12862421	12862422	DEL	MONO	tTTTTTTTTTTTTT
N2	N2_50	CHROMOSOME_II	1	13626403	13626404	DEL	MONO	aAAAAA
N2	N2_3	CHROMOSOME_X	1	17032785	17032785	SINS	MONO	aAAAAAAAA
N2	N-2	CHROMOSOME_I	1	3050224	3050225	DEL	MONO	aAAAAAAAAA
N2	N-2	CHROMOSOME_II	1	1372830	1372831	DEL	MONO	tTTTTTTTT
N2	N-2	CHROMOSOME_II	1	6800228	6800229	DEL	MONO	aAAAAAAAAAA
N2	N-4	CHROMOSOME_II	1	13626403	13626404	DEL	MONO	aAAAAA
N2	N-4	CHROMOSOME_III	1	10729931	10729932	DEL	MONO	tTTTTTTTT
N2	N-4	CHROMOSOME_III	1	11049705	11049706	DEL	MONO	tTTTTTTTT
N2	N-2	CHROMOSOME_III	1	13307901	13307902	DEL	MONO	tTTTTTTTTT
N2	N-4	CHROMOSOME_III	1	11764750	11764750	SINS	MONO	tTTTTTTTT
N2	N-4	CHROMOSOME_V	1	16781480	16781480	SINS	MONO	tTTTTTT
N2	N-4	CHROMOSOME_X	1	17032785	17032785	SINS	MONO	aAAAAAAAA
N2	N2_3	CHROMOSOME_II	1	8535067	8535067	SINS	MONO	aAAAAAAAAAAA
N2	N2_50	CHROMOSOME_X	1	8861877	8861878	DEL	MONO	tTTTTTTTTTTT
N2	N-4	CHROMOSOME_I	1	1264431	1264432	DEL	MONO	aAAAAAAAAAA
N2	N-4	CHROMOSOME_II	1	5020332	5020333	DEL	MONO	aAAAAAAAA
N2	N-4	CHROMOSOME_II	1	7188545	7188546	DEL	MONO	tTTTTTTTT
N2	N-4	CHROMOSOME_III	1	13307140	13307141	DEL	MONO	gGGGGGGGGGG
N2	N-4	CHROMOSOME_IV	1	16981105	16981106	DEL	MONO	aAAAAAAAAAA
N2	N2_3	CHROMOSOME_II	1	4642303	4642304	DEL	MONO	aAAA
polq-1	XF152_H50	CHROMOSOME_X	14	9225274	9225288	DEL	DI	agagagagagagagAGAGAGAGAGAGAGAGAGAGAGAGAGAGAGAGAGAG
polq-1	XF152_I50	CHROMOSOME_I	9	1546401	1546410	DEL	NINE	tcggcaaatTCGGCAAATTCGGCAAATTCGGCAAATTCGGCAAAT
polq-1	XF152_G50	CHROMOSOME_III	3	12566581	12566581	SINS	TRI	agaAGAAGAAGAAGA
polq-1	XF152_H50	CHROMOSOME_I	3	14124788	14124788	SINS	MONO	aaaAAAAAAAAAAAA
polq-1	XF152_H50	CHROMOSOME_V	2	11496752	11496754	DEL	MONO	ggGGGGGGGGGGGG
polq-1	XF152_I50	CHROMOSOME_III	2	5416080	5416082	DEL	MONO	ggGGGGGGGGGGGGG
polq-1	XF152_H50	CHROMOSOME_III	1	212206	212207	DEL	MONO	tTTTTTTTTTT
polq-1	XF152_G50	CHROMOSOME_X	1	2312399	2312399	SINS	MONO	cCCCCCCCCCC

polq-1	XF152_I50	CHROMOSOME_V	1	3449534	3449535	DEL	MONO	tTTTT
polq-1	XF152_I50	CHROMOSOME_IV	1	5199068	5199069	DEL	MONO	tTTTTTTT
polq-1	XF152_G50	CHROMOSOME_X	1	6580098	6580099	DEL	MONO	aAAAAAAAA
polq-1	XF152_E50	CHROMOSOME_V	1	8296059	8296060	DEL	MONO	aAAAAAAAA
polq-1	XF152_H50	CHROMOSOME_IV	1	8633324	8633325	DEL	MONO	tTTTTTTT
polq-1	XF152_E50	CHROMOSOME_I	1	10093794	10093795	DEL	MONO	aAAAAAAAAAA
polq-1	XF152_E50	CHROMOSOME_IV	1	13304597	13304598	DEL	MONO	aAAAAAAA
polq-1	XF152_E50	CHROMOSOME_X	1	16192386	16192387	DEL	MONO	aAAAAAAAAAA
polq-1	XF152_H50	CHROMOSOME_V	1	17052577	17052577	SINS	MONO	aAAAAAAAAAA
polq-1	XF152_I50	CHROMOSOME_V	1	17711073	17711074	DEL	MONO	aAAAAAAAAAA
polq-1	XF152_H50	CHROMOSOME_V	1	18421950	18421950	SINS	MONO	tTTTTTTTTTTTT
polq-1	XF152_E50	CHROMOSOME_X	1	15785644	15785645	DEL	MONO	gGGGGGGGGGG
